# 

**Published:** 1890-02-01

**Authors:** 


SATURDAY, FEBRUARY 1st, 1890.

**Life Seeds and Disinfection** - 271

**Words of Consolation**—The Dignity of Work - 272

**The Cyprus Society.** I.—Hospital Work - 273

**February** - 274

**The Doctor's Pulpit.**—Colour-Blindness - 275

**Popular Pastimes as Aids to Hospital Funds.** By A. W. WEBSTER - 276

**Everybody's Page** - 277

**Notes and News** - 278

**St. John's Hospital for Diseases of the Skin.**—Auditor's Report - 279

**Annotations.**—Doctors and their Degrees—Lotteries and Hospital Funds - 280

**The Practitioner's Outlook and Retrospect:**

MEDICINE—Albuminuria—Malingering—Aortic Aneurysm—Diagnosis of Sickness in the Street—Hypnotism - 281

Variations of Symptoms from the Type—Treatment of Ague - 282

SURGERY—Second Report of the Hyderabad Chloroform Commission - 282

Obstructed Breathing from Anæthesia - 283

DIETETICS—Monotonous Diet - 284

RECENT RESEARCH—Microbes - 284

**More Voyages in Vacation.**—II. - 284

**Fallow Crops.**—The Saturday Fund and St. John's Hospital 286

**Scraps and Gleanings** - 286

EXTRA SUPPLEMENT—THE NURSING MIRROR.

**En Passant.**—Ashton Nursing Association—Frome Nurses' Home—"The Nightingale"—More Nurses or Higher Pay—Mary Adelaide Letter—Central Sick Asylum—Sheffield Union—Thousands of Pounds for the National Pension Fund - lxxiii
**The Nurses' Bookshelf.**—Obstetric Nursing. II. - lxxiv
**In Days of Sickness.** By A PATIENT - lxxiv
**Examination Questions** - lxxv
**Everybody's Opinion.**—"Ladies and Workhouse Infirmaries"—The Pension Fund and Its Enemy—The Older Nurses - lxxv
**Appointments—Notices—Notes and Queries** - lxxvi
**Presentation—Vacancies** - lxxvi
**Amusements and Relaxation** - lxxvi

